# Targeting CAPON to modulate the CAPON–NOS Axis: a computational approach

**DOI:** 10.1016/j.csbj.2025.11.001

**Published:** 2025-11-04

**Authors:** Hossam Nada, Gerhard Wolber, Moustafa T. Gabr

**Affiliations:** a Department of Radiology, Molecular Imaging Innovations Institute (MI3), Weill Cornell Medicine, New York, NY 10065, USA; b Freie Universität Berlin, Molecular Design Group, Instute of Pharmacy, Department of Biology, Chemistry & Pharmacy, Königin-Luisestr. 2+4, Berlin 14195, Germany

**Keywords:** Drug Discovery, Small molecules, Virtual screening, Alzheimer's disease, Molecular dynamics

## Abstract

The carboxy-terminal PDZ ligand of neuronal nitric oxide synthase (CAPON) serves as a critical regulatory protein controlling nitric oxide (NO) signaling across multiple physiological and pathological processes which encompass neurological, cardiac and metabolic functions. These diverse physiological roles of CAPON marks it as a key therapeutic target for conditions associated with its dysregulation. Despite this therapeutic potential there are no specific CAPON or nNOS/CAPON modulators which have been developed to date, highlighting a significant gap in targeted drug discovery. Herein, we report the first strategy specifically focused on disrupting the nNOS/CAPON protein-protein interface. Through screening of a chemical library composed of 4.6 million compounds and 13 molecular dynamics simulations, nine potential hit compounds were identified. This work represents a foundational step toward developing targeted therapies for CAPON-mediated disorders.

Beyond identifying these promising hits, our approach introduces three python-based drug discovery tools: (i) a Python-based toolset for NMR structural analysis, clustering and visualization, (ii) accelerated ligand preparation toolkit, (iii) Automated hit prioritization pipeline based on multi-method consensus scoring approach that takes in account docking scores and MMGBSA. Collectively, these tools form an accelerated drug discovery pipeline that automates most of the virtual screening process and offers a scalable computational framework to support future drug discovery targeting protein–protein interactions.

## Highlights

• A novel structure-based strategy developed to target the CAPON/nNOS protein–protein interaction.
• A Python-based toolset for protein conformation analysis, identification, visualization, clustering and separation.
• Python pipeline enables efficient ligand preparation for ultra-large chemical libraries.
• A python pipeline for automated hit prioritization based on a multi-method consensus scoring approach.
• Consensus virtual screening identified 9 promising small-molecule candidates.

## Graphical abstract

**Figure:**
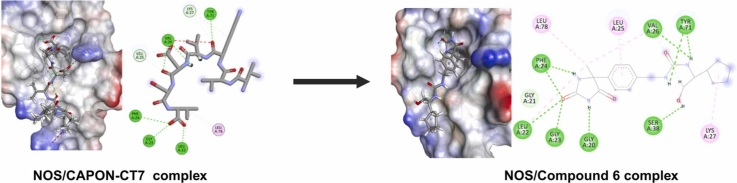

## 1 Introduction

The carboxy-terminal PDZ ligand of neuronal nitric oxide synthase (CAPON) is a key regulatory protein which is responsible for the modulation of nitric oxide (NO) signaling in various physiological and pathological processes. [1], [2] Nitric oxide synthase (NOS) exists in three distinct isoforms: neuronal-type (nNOS), inducible-type (iNOS), and endothelial-type (eNOS) [3]. Each of the NOS isoforms serve distinct specialized functions across different tissues including the cerebellum, skeletal muscles, kidneys, blood vessels, and immune cells [4], [5], [6]. Among the nine known proteins that interact with nNOS, CAPON is a crucial regulator of nNOS activity through its unique ability to compete with postsynaptic density protein 95 (PSD-95) for binding to the nNOS PDZ domain [7]. This competitive interaction fundamentally alters NO production and downstream signaling cascades, positioning CAPON as a master regulator of nitric oxide homeostasis.

**Fig. 1:**
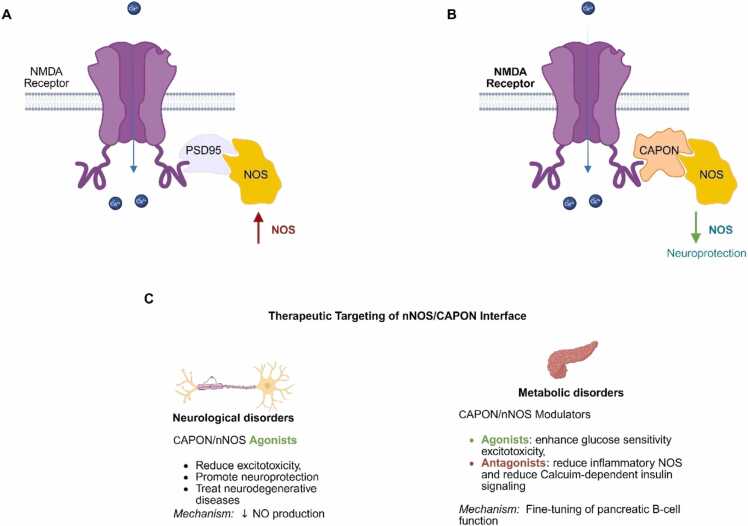
**CAPON-Mediated Modulation of NMDA Receptor–nNOS Signaling and Its Therapeutic Potential.** (A) Under normal physiological conditions, the activation of NMDA receptors lead to calcium influx which promotes the recruitment of PSD95 and neuronal nitric oxide synthase (nNOS), resulting in high nitric oxide (NO) production. This Ca²⁺-dependent NO signaling plays roles in neurotransmission but can also contribute to excitotoxicity and neuronal damage. (B) CAPON competitively binds to nNOS, displacing PSD95 and disrupting the NMDA receptor–nNOS signaling complex. This reduces NO production and confers neuroprotective effects by attenuating excitotoxicity. (C) Therapeutic strategies targeting the CAPON–nNOS interface include the use of agonists and modulators: In neurological disorders, CAPON/nNOS agonists reduce excitotoxicity and promote neuroprotection by decreasing NO production. In metabolic disorders, CAPON/nNOS modulation via agonists and antagonists are theorized to have different therapeutic effects. CAPON agonists will enhance glucose sensitivity and modulating excitotoxicity. Conversely, CAPON antagonists will reduce inflammatory NO production and restoring calcium-dependent insulin signaling.

The CAPON signaling pathway (Fig. 1) operates through a complex molecular switching mechanism that directly impacts neuronal function and cellular metabolism. Under normal conditions, nNOS forms a functional complex with N-methyl-D-aspartate (NMDA) receptors and PSD-95, facilitating calcium-dependent NO production in response to glutamatergic signaling [8]. However, CAPON disrupts this canonical NMDAR-PSD95-nNOS complex by competing for the same PDZ-binding motif on nNOS, resulting in the formation of an alternative NMDAR-CAPON-nNOS complex [2], [9]. This molecular rearrangement significantly attenuates nNOS activity and reduces NO production, thereby providing neuroprotection against excitotoxicity and oxidative stress. The downstream effects of CAPON-mediated nNOS regulation extend beyond the nervous system, influencing calcium channel function in cardiac myocytes [10] through S-nitrosylation of L-type calcium channels and modulating insulin signaling pathways [11], [12] in pancreatic cells. This multifaceted signaling network positions CAPON as a central hub connecting neuronal activity, cardiac electrophysiology, and metabolic regulation.

The therapeutic potential of CAPON is highly context-dependent due to its complex signaling mechanism. This is due to the diverse roles of CAPON which often necessitate opposing pharmacological approaches depending on the target tissue and disease context. In neurological disorders (Fig. 1C), the neuroprotective function of CAPON through nNOS modulation presents opportunities for treating neurodegenerative diseases and reducing excitotoxic neuronal damage. While in metabolic disorders, the enhancement of CAPON activity may improve pancreatic β-cell function and glucose sensitivity, while simultaneously risking the promotion of inflammatory pathways in other tissues. As such, the impact of CAPON is highly context-dependent and disease specific. Given the involvement of CAPON in multiple pathological conditions and signaling pathways, it has emerged as a compelling therapeutic target. In addition, the modulation of CAPON function offers not only a promising strategy for novel treatments but also a valuable avenue to deepen our understanding of its biological effects. Despite this potential, to the best of our knowledge no CAPON or nNOS/CAPON modulators have been developed to date which underscores the urgent and unmet need for targeted drug discovery in this space.

The conformational heterogeneity observed in nNOS is biologically significant and likely plays a crucial role in CAPON binding [13], [14]. Protein-protein interactions, particularly those involving disordered or flexible regions like the nNOS PDZ domain, frequently exploit conformational plasticity through conformational selection or induced-fit mechanisms. CAPON, which contains a C-terminal PDZ-binding motif [7], likely engages with multiple nNOS conformational states to achieve its regulatory function [15]. The presence of distinct conformational clusters in the NMR-derived ensemble suggests that nNOS exists in a dynamic equilibrium between multiple states, consistent with the conformational selection model wherein CAPON selectively binds and stabilizes specific conformations that favor interaction [16]. This plasticity may be essential for accommodating the CAPON binding motif and facilitating the formation of the nNOS-CAPON complex, which plays a critical role in neuronal signaling by anchoring nNOS to specific subcellular locations [17].

Despite the well-established therapeutic potential of disrupting the CAPON/nNOS interaction, this protein-protein interface has proven exceptionally challenging to target through conventional drug discovery approaches. One of the factors that has contributed to the difficulty of targeting the CAPON/nNOS interaction is due to the relatively flat binding surfaces and the reliance on backbone-mediated hydrogen bonds that are difficult to mimic with small molecules [18]. Another key factor hindering the targeting of the interaction interface is the high conformational flexibility of both the PDZ domain and the CAPON binding motif creates a moving target that complicates structure-based drug design efforts [19]. Herein we present an ensemble-based virtual screening approach which addresses these challenges by explicitly accounting for conformational heterogeneity, enabling the identification of compounds that can engage multiple nNOS conformational states and potentially achieve the selectivity and efficacy required for therapeutic intervention.

## 2 Results and discussion

### 2.1 NMR conformations analysis

The NMR structure of nNOS bound in complex with Asp–X–Val–COOH peptides of CAPON contains 15 distinct conformations. Analysis of the 15 nNOS conformations revealed significant structural diversity within the backbone atoms where the pairwise RMSD values ranging from 1.19 Å to 7.18 Å (mean: 3.42 ± 1.52 Å). The conformational analysis of the 1B8Q ensemble revealed substantial structural heterogeneity with pairwise backbone RMSD values spanning 1.187–7.180 Å (mean: 3.134 ± 1.503 Å) which indicates significant conformational plasticity across the 15-model ensemble. Unsupervised clustering with multi-metric optimization identified two distinct conformational states with robust statistical support (silhouette score [20]: 0.513, Davies-Bouldin index [21]: 0.311) which suggests a well-separated clusters with high internal cohesion and minimal inter-cluster ambiguity. The bimodal distribution comprises a dominant conformational basin (Cluster 0, 86.7 % occupancy, 13 models) and a minor alternative state (Cluster 1, 13.3 % occupancy, 2 models) which is consistent with the theorized two-state conformational equilibrium. Model 4 was identified as a consensus outlier across multiple orthogonal detection methods (IQR-based [22], Isolation Forest [23], and hierarchical singleton analysis), likely representing either a transient intermediate state, an improperly equilibrated structure, or a computational artifact. The convergence of three independent outlier detection algorithms provides high confidence for excluding this model from downstream applications, as its inclusion could introduce spurious conformational states that do not reflect the native ensemble landscape.

**Fig. 2:**
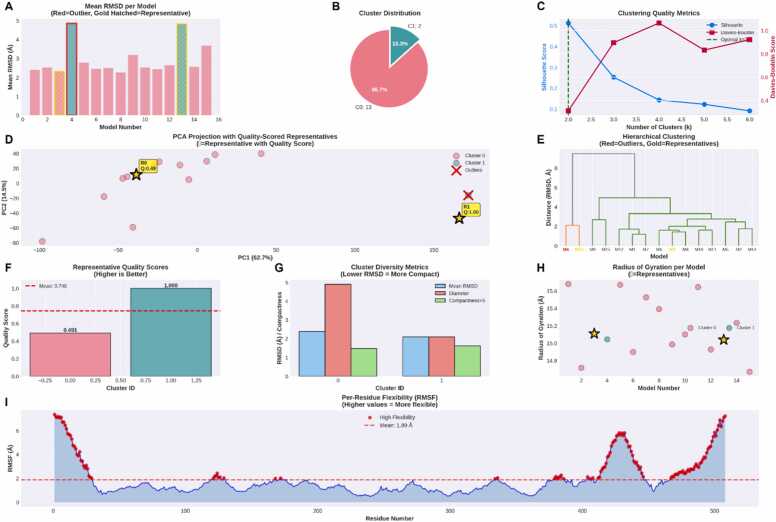
**Comprehensive conformational analysis of the 1B8Q ensemble with quality-scored representative selection. A)** Mean backbone RMSD of each model to all other structures in the ensemble. Red outlines indicate consensus outliers flagged by multiple detection methods; gold hatched bars with borders denote cluster representatives selected via multi-method consensus. **B)** Cluster membership distribution showing the relative proportion of models assigned to each conformational cluster. **C)** Clustering quality metrics across different k values. Blue circles represent silhouette scores (left y-axis, higher is better), red squares show Davies-Bouldin scores (right y-axis, lower is better), and the green dashed line indicates the optimal k = 2 selected based on silhouette score maximization. **D)** Principal component analysis (PCA) projection displaying the first two principal components (PC1: 62.7 %, PC2: 14.5 %). **E)** Hierarchical clustering dendrogram using Ward's linkage on the pairwise RMSD matrix. Red labels identify outliers, gold labels mark representatives, and black lines delineate cluster boundaries. **F)** Representative quality scores computed as weighted composite metrics (35 % average RMSD, 25 % maximum RMSD, 25 % coverage, 15 % centrality). **G)** Cluster diversity metrics displaying intra-cluster mean RMSD (blue), cluster diameter (red), and compactness× 5 (green) for each cluster. Lower RMSD values indicate more structurally homogeneous clusters. **H)** Radius of gyration (Rg) distribution across models, color-coded by cluster assignment. Gold stars mark representative structures. **I)** Per-residue root mean square fluctuation (RMSF) across the backbone, with red markers highlighting high-flexibility regions exceeding the 75th percentile (red dashed line indicates ensemble mean: 1.89 Å).

Representative selection (Fig. 2) using a multi-method consensus approach (integrating medoid, centroid, minimax, and density peak algorithms) identified Model 3 as the Cluster 0 representative with a composite quality score of 0.491, while Model 13 achieved a perfect score (1.000) as the Cluster 1 representative. The moderate quality score for Model 3 reflects the inherent structural diversity within the dominant cluster (average intra-cluster RMSD: 1.81 Å, coverage: 61.5 %), indicating that no single structure can perfectly represent all conformational substates within this broad basin. This 61.5 % coverage metric reveals that only approximately two-thirds of cluster members lie within 2 Å RMSD of the representative, suggesting residual conformational heterogeneity that may be functionally relevant. In contrast, while Cluster 1 biological validity was strongly supported by the substantial inter-representative RMSD of 5.623 Å, confirming genuine conformational divergence rather than statistical noise.

The spatial distribution of conformational flexibility revealed by per-residue RMSF analysis provides mechanistic insights into the structural dynamics underlying the observed clustering pattern. Three distinct high-mobility regions exhibit RMSF values exceeding 6 Å: the N-terminal region (residues 1–50), a central loop domain (residues 250–300), and the C-terminal tail (residues 450–550). These flexible regions likely serve as conformational hinges that facilitate transitions between the two identified cluster states, with their elevated mobility potentially correlating with functional mechanisms such as substrate binding, allosteric regulation, or domain-domain interactions. The two-dimensional PCA projection captures 77.2 % of total variance (PC1: 62.7 %, PC2: 14.5 %), demonstrating that the conformational landscape is effectively low-dimensional and well-represented by the principal modes of motion. The clear spatial separation between clusters in PC space, combined with the moderate-to-high silhouette scores, indicates that the clustering solution genuinely reflects the underlying conformational distribution rather than arbitrary partitioning. The cluster compactness values (0.295 for Cluster 0, 0.323 for Cluster 1) are relatively similar, suggesting comparable internal cohesion despite the dramatic difference in cluster size, which further validates the two-state model as an appropriate description of the ensemble architecture.

Accordingly, for structure-based drug design and ensemble docking applications both Models 3 and 13 can be employed to capture the bimodal conformational distribution while maintaining computational tractability. The substantial inter-representative separation (5.623 Å) ensures comprehensive sampling of distinct conformational states, which is critical for accurately modeling protein-ligand interactions in systems where backbone flexibility modulates binding site geometry, accessibility, or pharmacophore presentation. The exclusion of outlier Model 4 is essential to prevent contamination of binding pose predictions with non-native conformations that could lead to false positives in virtual screening or erroneous structure-activity relationship interpretations. The clustering metrics collectively that the models 3 and 13 adequately capture the primary conformational modes.

### 2.2 Library preparation

Ligand preparation for ultra-large libraries is a computationally intensive process which demands substantial computational storage and memory resources. [24], [25] The Enamine Screening Collection (4.6 million compounds) was chosen for the screening efforts in this work due to its extensive chemical diversity, drug-like properties and its proven track record for success high-throughput virtual screening campaigns [26], [27], [28]. The library offers a broad representation of lead-like and fragment-like molecules, making it a suitable source for identifying potential hits with favorable pharmacokinetic and physicochemical characteristics. Initial ligand preparation efforts revealed that processing these libraries on our system which is equipped with an Nvidia 470 Ti GPU and 32 CPU cores would require high computational cost and time. To address this bottleneck, a streamlined ligand preparation pipeline (Supplementary folder) was developed to significantly reduce computational overhead and accelerate the processing time. The ligand preparation pipeline implemented here offers a reproducible, scalable, and chemically sound approach to generating screening-ready small molecules. The process incorporates a robust suite of standardization and filtering criteria commonly accepted in early-stage drug discovery. The pipeline’s use of RDKit [29] for SMILES parsing, descriptor calculation, and molecular embedding ensures chemical accuracy and compatibility with cheminformatics standards.

Notably, the parallelized processing of molecules using Python’s multiprocessing tools dramatically increases throughput, making the pipeline suitable for libraries containing tens of thousands of compounds. The integration of multiple structural alert filters, including PAINS [30] and BRENK [31], enhances the downstream screening quality by removing promiscuous and toxic scaffolds. The charge neutralization step is especially valuable for virtual screening workflows where charged ligands may show artificial binding due to force field artifacts.

Additionally, the pipeline’s modular design allows for user customization at each stage via command-line arguments (e.g., enabling 3D coordinate generation, skipping Lipinski rules, or keeping duplicates). Comprehensive logs generated at each step offer transparency, facilitate troubleshooting, and allow auditing of filtered-out compounds with rationales. Finally, by packaging the pipeline with setup scripts and standardized dependencies (as seen in setup_package.py), it can be easily installed and shared across research teams, ensuring reproducibility and ease of deployment.

### 2.3 Virtual screening results

To identify potential compounds capable of disrupting CAPON/NOS binding, the two prepared compound libraries from Enamine were subjected to a consensus virtual screening workflow targeting the identified CAPON/NOS binding site using the two representative conformations (models 3 and 13). The virtual screening workflow involved the Enamine Screening Collection which comprised of 4.6 million readily available compounds. The virtual screening protocol was executed in three sequential stages using the GLIDE module of Schrodinger which involved High-Throughput Virtual Screening (HTVS), Standard Precision (SP), and Extra Precision (XP) docking of the prepared libraries. In this workflow a stepwise filtration approach was employed in which only the top 10 % of compounds from each stage advanced to the next stage.

**Fig. 3:**
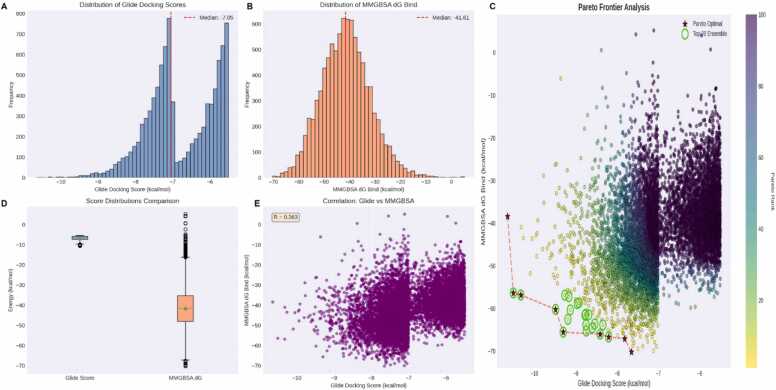
**Distribution and correlation analysis of molecular docking scores.** (A) Histogram showing the distribution of Glide docking scores across 9353 hits with median value indicated by the red dashed line at −7.05 kcal/mol. (B) Distribution of MMGBSA dG Bind values with median at −41.61 kcal/mol, demonstrating broader variability compared to docking scores. (C) Pareto frontier analysis highlighting optimal binding solutions. Scatter plot of the hits colored by Pareto rank, where compounds closer to the bottom-left represent superior binding energetics. Red stars mark the nine Pareto-optimal compounds (rank 1) that define the non-dominated frontier, connected by a red dashed line. Green circles with black borders highlight the top 20 ensemble-ranked compounds, demonstrating strong enrichment near the Pareto frontier. (D) Box plot comparison of Glide and MMGBSA score distributions, with box boundaries representing the interquartile range, whiskers extending to 1.5 × IQR, and green triangles indicating mean values. (E) Scatter plot illustrating the correlation between Glide docking scores and MMGBSA dG Bind values (R = 0.36, n = 9353), revealing moderate positive correlation and complementary information captured by both scoring metrics.

An ensemble consensus scoring pipeline was developed and implemented in Python utilizing five complementary ranking algorithms (z-score normalization, percentile ranking, weighted averaging with 0.4/0.6 Glide/MMGBSA weighting, Pareto frontier analysis, and strict dual-cutoff filtering) to prioritize 9353 virtual screening hits which ultimately identified nine Pareto-optimal compounds that occupy the non-dominated frontier by simultaneously optimizing both Glide docking scores (≤ −9.49 kcal/mol) and MMGBSA dG Bind values (≤ −60.29 kcal/mol). The correlation analysis revealed a moderate positive correlation (R = 0.363) between Glide docking scores and MMGBSA dG Bind values which indicates that while both metrics assess binding affinity, they capture complementary aspects of protein-ligand interactions (Fig. 3). The distribution analysis showed that docking scores ranged from −10.65 to −5.50 kcal/mol (mean: −6.80 ± 0.91 kcal/mol), while MMGBSA calculations exhibited greater variability, spanning −70.27–5.17 kcal/mol (mean: −41.47 ± 9.65 kcal/mol). This broader distribution in MMGBSA scores reflects its enhanced sensitivity to electrostatic and solvation contributions, making it particularly valuable for discriminating high-quality binders.

**Fig. 4:**
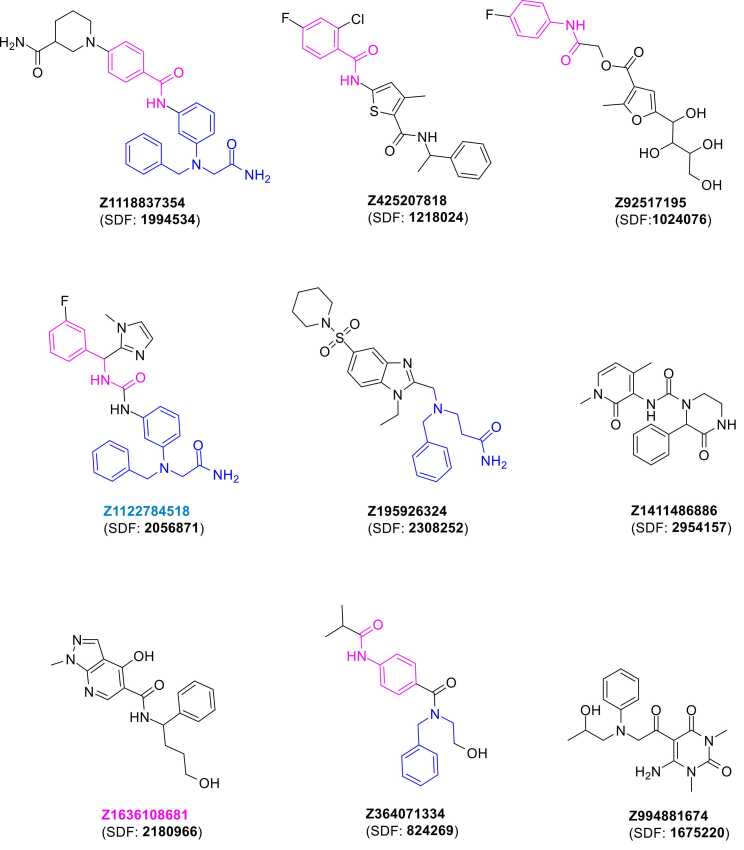
**Two-dimensional structures of the top nine compounds prioritized as predicted hits through consensus virtual screening and hit prioritization analysis.** key structural similarities highlighted to illustrate common pharmacophore features.

Notably, 4269 compounds (45.6 %) passed the strict dual cutoffs (docking score ≤ −6.0 kcal/mol and MMGBSA dG Bind ≤ −40.0 kcal/mol), indicating a substantial proportion of the library demonstrated favorable binding characteristics by both metrics. The Pareto frontier analysis identified nine compounds occupying the optimal trade-off surface between docking and MMGBSA scores, representing non-dominated solutions where improvement in one metric cannot be achieved without compromising the other. The top nine hits (Fig. 4) identified by the consensus docking scores were chosen to be validated via MD assays.

**Fig. 5:**
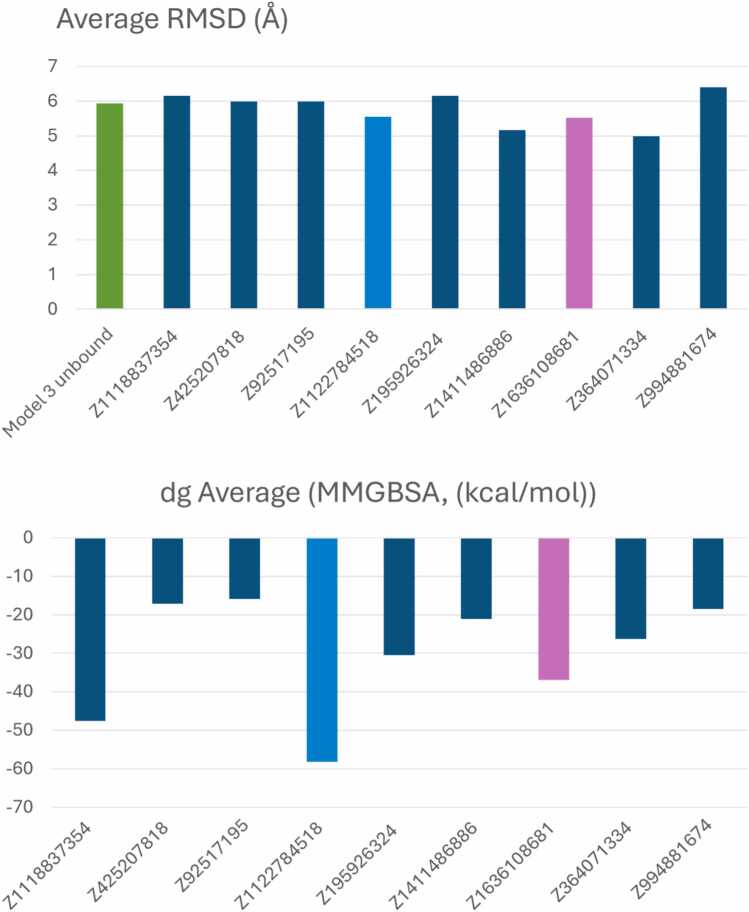
**Structural stability and binding affinity analysis of nNOS-ligand complexes.** (A) Average RMSD values calculated over the 200 ns of the MD simulation trajectory for unbound nNOS Model 3 (green) and complexes with the top 9 hit compounds. Lower RMSD values indicate enhanced structural stability. (B) Binding free energies (ΔG) calculated using MM-GBSA for each ligand-nNOS complex. More negative values indicate stronger binding affinity.

Subsequently, ten 200 ns MD simulations were performed to compare the binding affinities of the top 9 hits against unbound Model 3 of nNOS. The MD simulations revealed that not all hits were able to stabilize the nNOS protein., with average RMSD values ranging from 4.9 to 6.4 Å (Fig. 5A). The unbound Model 3 exhibited an average RMSD of 5.9 Å, serving as the reference for structural dynamics. Notably, compounds **Z1122784518** and **Z1696108681** demonstrated enhanced structural stability with RMSD values of 5.50 Å each, indicating minimal structural perturbation upon binding and suggesting stable complex formation throughout the simulation period.

MM-GBSA calculations revealed that Z1122784518 and Z1696108681 emerged as the most promising candidates, combining both structural stability and favorable binding energetics (Fig. 5B). **Z1122784518** exhibited the strongest binding affinity with ΔG of −58.7 kcal/mol, while Z1696108681 demonstrated ΔG of −37.2 kcal/mol. All tested compounds demonstrated negative binding free energies ranging from −15.6 to −58.7 kcal/mol, confirming favorable protein-ligand interactions. The convergence of enhanced structural stability and strong binding affinity positions **Z1122784518** as the lead candidate, with Z1696108681 serving as a promising secondary hit for further experimental validation. Conversely, despite exhibiting optimal structural stability (RMSD = 4.99 Å), **Z364071334** was eliminated from further consideration due to its comparatively weak binding affinity (ΔG = −26.28 kcal/mol) and the presence of unstable protein-ligand interactions (Supplementary file).

**Fig. 6:**
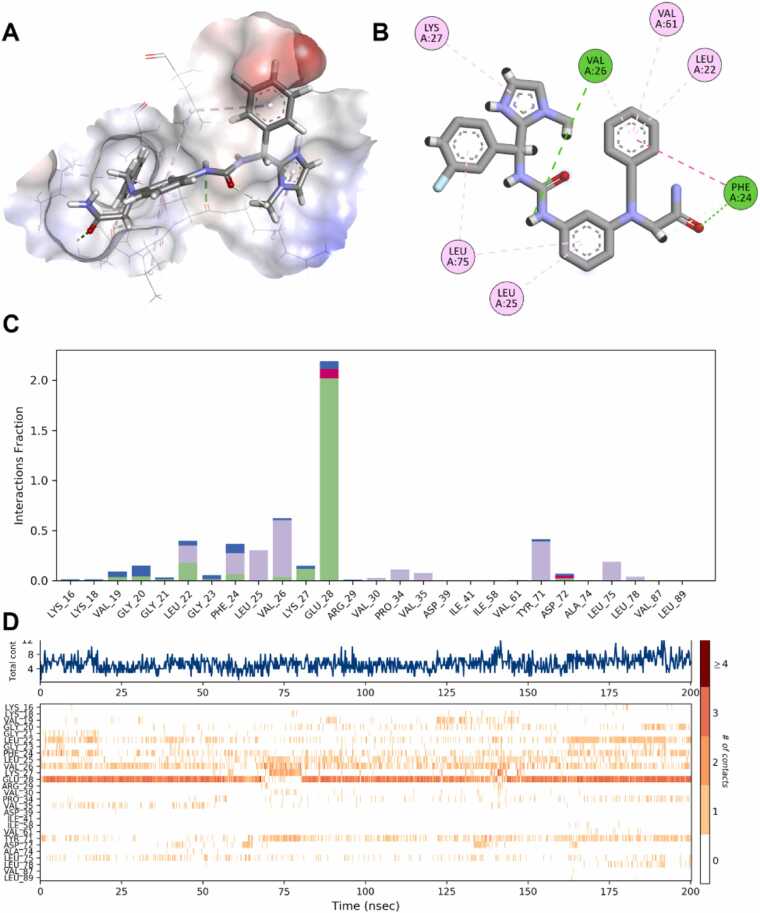
**Molecular dynamics analysis of Z1122784518 binding to nNOS Model 3.** (A) Three-dimensional representation of **Z1122784518** bound within the nNOS active site pocket. (B) Two-dimensional interaction diagram showing **Z1122784518** and its surrounding residues. (C) Interaction fraction analysis across the 200 ns MD trajectory. Bar heights represent the fraction of simulation time each residue-maintained contact with the ligand, color-coded by interaction type (green: hydrogen bonds, purple: hydrophobic contacts, blue: water bridges, red: ionic interactions). (D) Contact timeline heatmap showing the temporal dynamics of protein-ligand interactions.

Given the promising results of **Z1122784518** and **Z1696108681**, deeper analysis was warranted. Analysis of the docking complex of **Z1122784518** in complex with nNOS (Fig. 6A-B) demonstrated that Z1122784518 occupied the active site pocket with several interactions involving multiple residues. The ligand established hydrogen bonds with Val 26 and Phe 24 (green dashed lines), while forming hydrophobic contacts with Lys 27, Leu 22, Val 61, Leu 75 and Leu 25 (pink dashed lines). Interaction fraction analysis (Fig. 6C) revealed that Gly 29 maintained the most persistent contact via a hydrogen bond throughout the 200 ns of the MD simulation (>2.0 fraction), with additional stable interactions observed for Val 26, Leu 25, and Lys 27. The contact timeline (Fig. 6D) confirmed sustained protein-ligand interactions across the entire simulation period, with the total contact count remaining relatively constant at approximately 4–6 contacts. Notably, residues Val 26, Leu 25, and Phe 24 exhibited continuous engagement while other residues demonstrated more dynamic interaction patterns. These findings, combined with the favorable binding free energy of −58.7 kcal/mol and low RMSD of 5.5 Å, support **Z1122784518** as a stable and high-affinity nNOS inhibitor candidate.

**Fig. 7:**
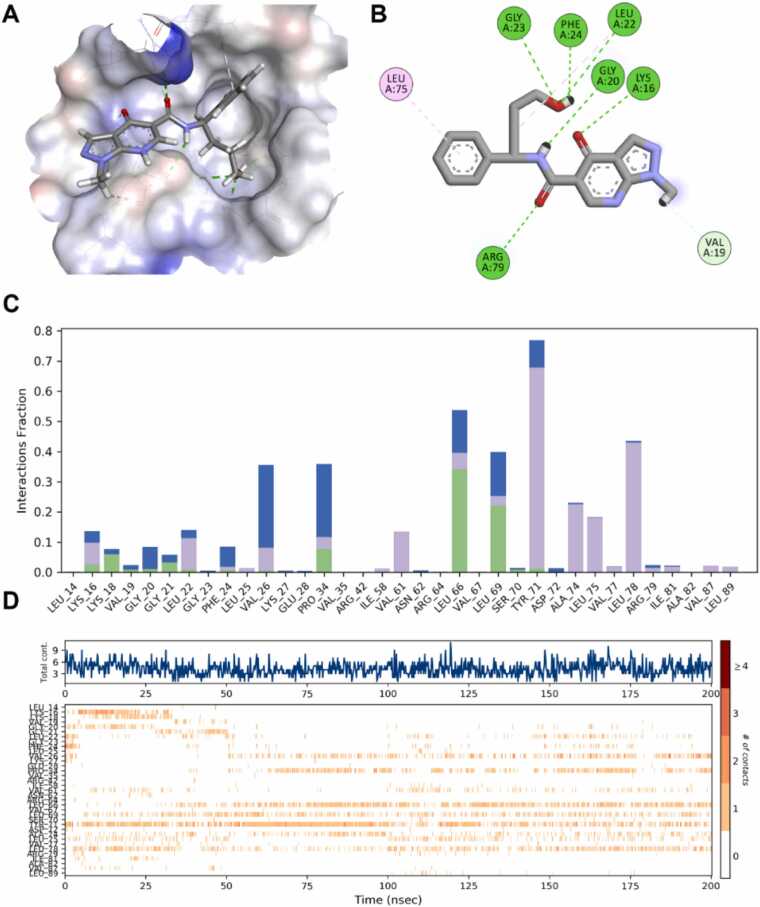
**Molecular dynamics analysis of Z1696108681 binding to nNOS Model 3.** (A) Three-dimensional representation of **Z1696108681** bound within the nNOS active site pocket. (B) Two-dimensional interaction diagram showing **Z1696108681** and its surrounding residues. (C) Interaction fraction analysis across the 200 ns MD trajectory. (D) Contact timeline heatmap showing the temporal dynamics of protein-ligand interactions.

MD simulations of the **Z1696108681**-nNOS complex demonstrated robust binding stability over the 200 ns trajectory with a distinct interaction profile compared to **Z1122784518**. The binding mode analysis (Fig. 7A-B) showed that **Z1696108681** positioned itself within the active site with hydrogen bonding interactions involving Lys 16, Gly 20, Leu 22, Gly 23, Phe 24 and Arg 79 (green dashed lines), while establishing hydrophobic contacts with Leu 75 and Val 19. Interaction fraction analysis (Fig. 7C) revealed Tyr 71 as the most persistent interacting residue, followed by Leu 66 and Leu 69, indicating a binding orientation different from that of **Z1122784518**. The contact timeline (Fig. 7D) demonstrated sustained protein-ligand engagement throughout the simulation, with total contact counts fluctuating between 3 and 9 contacts. Multiple residues, including Leu66, Tyr 71 and Leu 78 exhibited persistent contact patterns while others displayed more transient interactions. These findings, combined with the favorable binding free energy of −37.2 kcal/mol and optimal RMSD of 5.5 Å, establish **Z1696108681** as a promising secondary hit with complementary binding characteristics to **Z1122784518**.

**Fig. 8:**
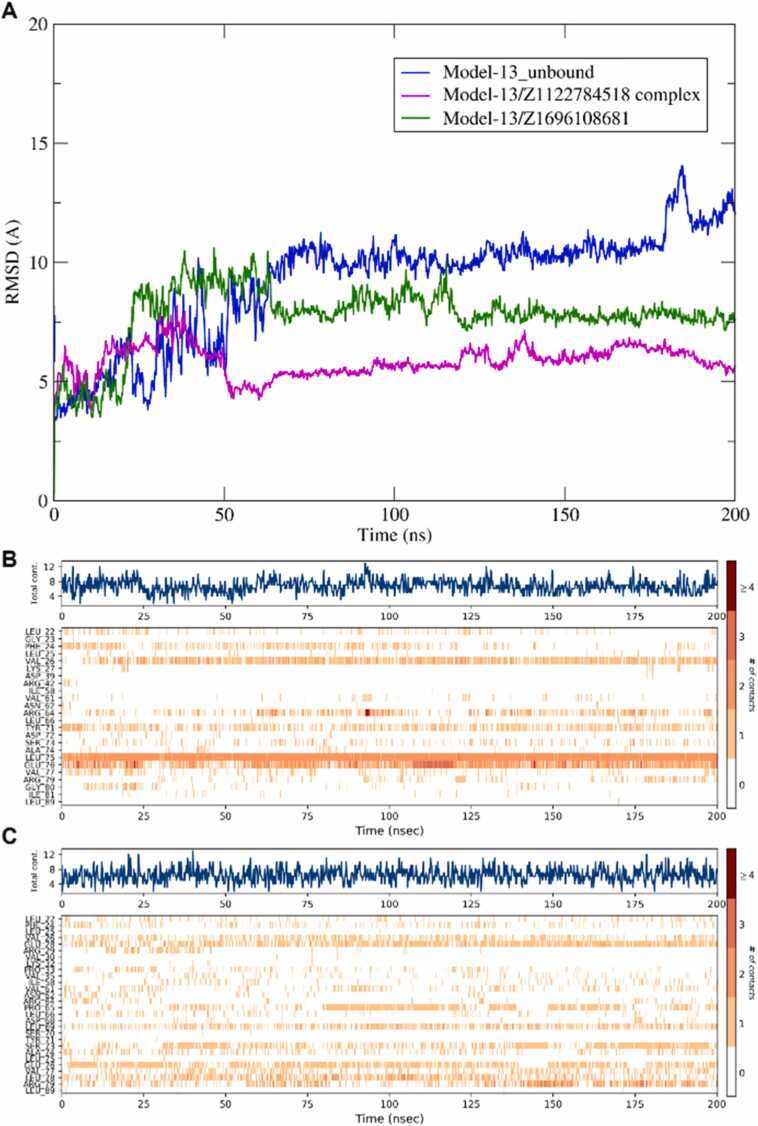
**Molecular dynamics simulation of top hit compounds bound to nNOS Model 13.** (A) RMSD trajectories over 200 ns for unbound Model 13 (blue), Model 13 in complex with Z1122784518 (magenta), and Model 13 in complex with Z1696108681 (green). (B) Contact timeline heatmap for the Z1122784518-Model 13 complex. (C) Contact timeline heatmap for the Z1696108681-Model 13 complex.

To validate the binding stability of the top two hits across different nNOS conformations, **Z1122784518** and **Z1696108681** were subjected to 200 ns MD simulations in complex with Model 13, the representative structure of the second most populated conformational cluster. RMSD analysis (Fig. 8A) revealed that both ligand-bound complexes exhibited enhanced structural stability compared to the unbound Model 13. The **Z1122784518**-Model 13 complex (magenta) maintained the lowest RMSD throughout the simulation with an average RMSD of 5.85 Å. Meanwhile, the **Z1696108681**-Model 13 complex (green) showed moderate stability with an average RMSD of 7.77 Å. In contrast, the unbound Model 13 (blue) displayed significantly higher structural fluctuations, with RMSD values escalating from 10 Å to 14 Å after 175 ns with an average RMSD of 9.18 Å which suggests inherent conformational instability. Contact timeline analysis for Z1122784518 (Fig. 8B) demonstrated persistent interactions with key residues including Val26, Leu 75 and Glu 76throughout the trajectory, with total contact counts remaining stable at 4–8 interactions. Similarly, Z1696108681 (Fig. 8C) maintained consistent engagement with residues such as Glu28, Glu76, Leu 78 and Arg79 with comparable contact counts of 4–8 interactions across the simulation. These findings confirm that both compounds stabilize the two representative conformation of nNOS (3 and 13) which supports their potential as robust CAPON/nNos modulators.

## 3 Methodology

### 3.1 NMR conformations analysis

The 1B8Q multi-model ensemble (15 structures) was analyzed using python analysis toolkit (Supplementary file) to quantify backbone structural heterogeneity and identify representative conformations for ensemble-based docking applications. All models were superimposed onto a reference frame (Model 1) using least-squares fitting of backbone atoms (Cα, C, N, O) within chain A to minimize translational and rotational differences. Following alignment, pairwise root mean square deviation (RMSD) matrices were computed across all 508 backbone atoms for each model pair, generating a comprehensive 15 × 15 distance matrix that captured the complete conformational landscape. Additional structural metrics were calculated including per-model radius of gyration (Rg), per-residue root mean square fluctuation (RMSF), and global mean RMSD to quantify both individual model compactness and ensemble-wide flexibility patterns.

Conformational outliers were identified using a consensus approach integrating three independent methods: (i) interquartile range (IQR) analysis on mean RMSD distributions (threshold: Q1 - 1.5 ×IQR to Q3 + 1.5 ×IQR), (ii) Isolation Forest algorithm applied to flattened coordinate matrices (contamination parameter: 0.1), and (iii) hierarchical clustering with Ward's linkage to detect singleton clusters at μ + 2σ distance thresholds. Models flagged by at least two methods were designated consensus outliers and excluded from representative selection. Optimal cluster number (k) was determined by evaluating k = 2–6 using multiple quality metrics on non-outlier structures: silhouette coefficient (maximized), Davies-Bouldin index (minimized), Calinski-Harabasz score (maximized), and elbow detection on within-cluster sum of squares (inertia). Final clustering was performed using k-means with k = 2, initialized with 20 independent runs to ensure convergence to the global optimum. Cluster quality was validated through silhouette analysis, and intra-cluster diversity was quantified using mean pairwise RMSD, cluster diameter (maximum pairwise distance), and compactness scores.

Representative structures for each cluster were identified using a consensus voting scheme integrating four independent selection criteria: (i) medoid selection (minimum average RMSD to cluster members), (ii) centroid proximity (minimum distance to geometric mean coordinates), (iii) minimax optimization (minimum of maximum pairwise RMSD), and (iv) density peak detection (maximum neighbor count within the 25th percentile RMSD threshold). The most frequently selected model across methods was designated the primary representative. Representative quality was quantified using a composite scoring function weighted as follows: 35 % normalized average RMSD (lower is better), 25 % normalized maximum RMSD (lower is better), 25 % coverage (percentage of cluster members within 2 Å), and 15 % centrality (inverse distance rank within cluster). Inter-representative RMSD was calculated to assess conformational space coverage, and principal component analysis (PCA) was performed on flattened coordinate matrices to visualize cluster separation in reduced dimensionality space. All analyses were implemented in Python 3.9 using scikit-learn v1.0 for clustering and dimensionality reduction, SciPy v1.7 for hierarchical methods, and custom algorithms for quality scoring.

### 3.2 Library preparation

The ligand preparation process was performed using a modular and automated Python pipeline implemented in the chem_pipeline.py script (Supplementary file), designed to handle high-throughput molecular preprocessing. Initially, raw ligand structures provided in a delimited text or CSV file were processed using the pipeline’s CSV preparation module. This module automatically detected and standardized the delimiter format (e.g., commas, tabs, or semicolons), ensuring robust compatibility across diverse datasets. Following standardization, the clean CSV was parsed to extract SMILES strings and associated compound IDs, which were then converted into 2D or optionally 3D structure-data file (SDF) representations using RDKit. Error handling and logging were integrated at this stage to discard malformed or non-interpretable SMILES strings.

The resulting SDF was subjected to a comprehensive filtering stage that applied a hierarchy of drug-likeness and safety criteria. Molecules were processed in parallel using multiple CPU cores to accelerate throughput. Filtering included enforcement of Lipinski’s Rule of Five [32], [33], additional drug-likeness constraints such as topological polar surface area (TPSA), number of rotatable bonds, and fraction of sp³ hybridized carbons, and substructure flags based on PAINS, BRENK, and NIH structural alerts. Canonical tautomers were generated, and molecules were standardized, uncharged, and optionally kekulized and hydrogenated. Molecules failing any criterion were filtered out, and detailed logs were saved for traceability. In the final stage, molecules with non-zero formal charges at physiological PH were identified and removed using a charge screening step, yielding a final SDF file composed exclusively of structurally and physiochemically suitable, neutral ligands. Duplicate structures were eliminated based on canonical SMILES unless explicitly retained by user configuration.

### 3.3 SBVS

Structure-based virtual screening was applied to identify potential CAPON/NOS binding possible modulators from the Enamine compound libraries using a hierarchical docking approach. The NOS protein structure was obtained from the Protein Data Bank (PDB ID: 1B9Q [15]) and prepared for virtual screening using the Protein Preparation Wizard in Schrödinger Suite. The CAPON/NOS binding site was identified and validated through analysis of the co-crystal structure, with receptor grids generated using Glide's grid generation module to represent the shape and electrostatic features of the binding pocket through multiple separate field sets. The consensus virtual screening protocol employed a three-stage hierarchical filtering approach using Glide's High-Throughput Virtual Screening (HTVS), Standard Precision (SP), and Extra Precision (XP) docking modes of the two representative models (models 3 and 13). Initially, the 4.6 million compounds from the Enamine Screening Collection were subjected to HTVS docking. Only the top 10 % of compounds from each stage were advanced to the subsequent precision level, ensuring computational efficiency while maintaining screening accuracy. Glide managed conformational flexibility through thorough conformational search followed by rapid exclusion of unfavorable conformations, with ligand poses scored progressively using increasingly accurate algorithms.

A multi-method consensus scoring approach was employed to prioritize the hits from the virtual screening where glide docking scores and MM-GBSA binding free energies were integrated. Five distinct ranking strategies were implemented: (1) Z-score normalization with equal weighting, where both metrics were standardized to zero mean and unit variance before averaging; (2) percentile-based ranking, which converts raw scores to percentile ranks (0−100) to provide robustness against outliers; (3) weighted Z-score averaging, assigning 60 % weight to MM-GBSA and 40 % to Glide scores based on the typically higher accuracy of MM-GBSA calculations; (4) Pareto optimal ranking, identifying non-dominated solutions where no other hits performs better in both metrics simultaneously, with iterative removal of non-dominated frontiers to assign hierarchical ranks; and (5) strict cutoff filtering, applying thresholds of ≤ -6.0 kcal/mol for Glide gscore and ≤ -40.0 kcal/mol for MM-GBSA dG Bind, followed by Z-score ranking of compounds passing both criteria. An ensemble ranking was calculated by averaging the ranks obtained from all five methods, providing a robust consensus that minimizes bias from any single scoring function. Compounds with missing values in either metric were excluded from analysis. The final prioritization list was generated by ranking compounds according to their ensemble scores, with lower ranks indicating superior predicted binding affinity. This multi-method approach ensures that top-ranked compounds demonstrate consistent excellence across diverse scoring algorithms, reducing false positive rates inherent in single-metric screening and providing a more reliable selection of candidates for experimental validation.

### 3.4 Molecular docking study

Following the hierarchical screening, compounds that passed the final XP filtering stage were subjected to a molecular docking analysis to predict their binding modes and affinities to the CAPON/NOS binding site. Visual inspection of binding orientations to ensure reasonable protein-ligand interactions and full occupancy was employed to select the most promising hits for further analysis. Molecular visualization and analysis were performed using BIOVIA Discovery Studio Visualizer to assess binding mode feasibility and identify critical pharmacophoric interactions.

### 3.5 Molecular dynamics study

To validate the stability of predicted binding poses and investigate protein conformational dynamics, molecular dynamics simulations were conducted on the most promising hits. A total of 13 independent 200 ns MD simulations were performed using Desmond molecular dynamics package. All systems were solvated in explicit TIP3P water molecules within periodic boundary conditions, with appropriate counter-ions added to maintain system neutrality. The OPLS3e force field was employed for protein and ligand parameterization, with simulations conducted at 300 K and 1 atm pressure using NPT ensemble conditions. Root Mean Square Deviation (RMSD) values were calculated for protein backbone atoms throughout the simulation trajectories to assess binding pose stability and protein conformational changes, with the initial docked structure serving as the reference frame for RMSD calculations. The protein-ligand interactions were analyzed using the Protein Interaction Wizard within DEMSOND. RMSD plots were visualized using the XmGrace software.

## 4 Limitations and future directions

While our computational approach has identified promising small-molecule candidates targeting the nNOS/CAPON interface, several important limitations must be addressed. Most critically, these findings require rigorous experimental validation to confirm binding affinity, selectivity, and functional activity in biological systems. Additionally, the predicted hits carry inherent uncertainty regarding their physiological effects due to CAPON's dual role in both promoting and regulating nNOS activity across different tissue contexts and pathological conditions. The complex nature of CAPON-mediated signaling pathways means that disrupting the nNOS/CAPON interaction could yield context-dependent outcomes that computational models cannot fully predict. To address these limitations and advance toward therapeutic development, we are currently developing a NanoBRET (Bioluminescence Resonance Energy Transfer) assay that will enable real-time quantification of how these candidate modulators affect the nNOS/CAPON protein-protein interaction in living cells. This experimental validation platform will be essential for translating our computational discoveries into viable therapeutic candidates and for understanding the nuanced biological consequences of pharmacologically modulating this critical signaling axis.

## 5 Conclusions

Our consensus virtual screening campaign evaluated 4.6 million compounds to identify novel small molecule modulators of the CAPON/nNOS protein-protein interaction. The virtual screening predicted 9 promising candidates that were subsequently validated using 200 ns molecular dynamics simulations. Comparative analysis against both the unbound protein and the CAPON/NOS complex revealed two lead compounds demonstrating superior binding stability, sustained molecular contacts, and energetically favorable binding conformations. To assess binding robustness across conformational states, the top two hits were subjected to MD simulations with the second cluster representative (Model 13). Both compounds exhibited stabilizing effects on Models 3 and 13, confirming their potential as effective CAPON/nNOS modulators across diverse protein conformations. These computationally validated hits represent high-priority candidates for future experimental validation.

Beyond the identification of potential therapeutic leads, this work introduces three Python-based toolkits for protein structure analysis and streamlined ligand preparation. The NMR analysis toolkit enables rapid processing and visualization of structural data, along with conformational clustering to streamline virtual screening workflows. Meanwhile, the ligand preparation workflow offers an automated solution to rapidly prepare ligands. Lastly, the hit prioritization toolkit integrates both binding free energy and docking scores to rank virtual screening hits. Together, these toolkits significantly reduce computational overhead and processing time compared to conventional approaches, thereby enhancing efficiency in large-scale virtual screening while minimizing bias in hit selection. Collectively, these findings establish a solid foundation for the rational design of CAPON/nNOS modulators and provide valuable computational tools that will accelerate future drug discovery efforts targeting this therapeutically relevant protein-protein interaction.

## CRediT authorship contribution statement

**Gabr Moustafa:** Supervision, Funding acquisition. **Hossam Nada:** Writing – original draft, Validation, Methodology, Investigation, Formal analysis, Data curation, Conceptualization. **Gerhard Wolber:** Writing – review & editing, Supervision.

## Ethics Statement

Not applicable.

## Declaration of Competing Interest

The authors declare that they have no known competing financial interests or personal relationships that could have appeared to influence the work reported in this paper.
