# Supplementary figures and images for: Targeting CAPON to modulate the CAPON–NOS Axis: a computational approach
Source: Comput Struct Biotechnol J. 2025 Nov 4;27:4813–24. doi: 10.1016/j.csbj.2025.11.001 (PMC12636374; doi:10.1016/j.csbj.2025.11.001)

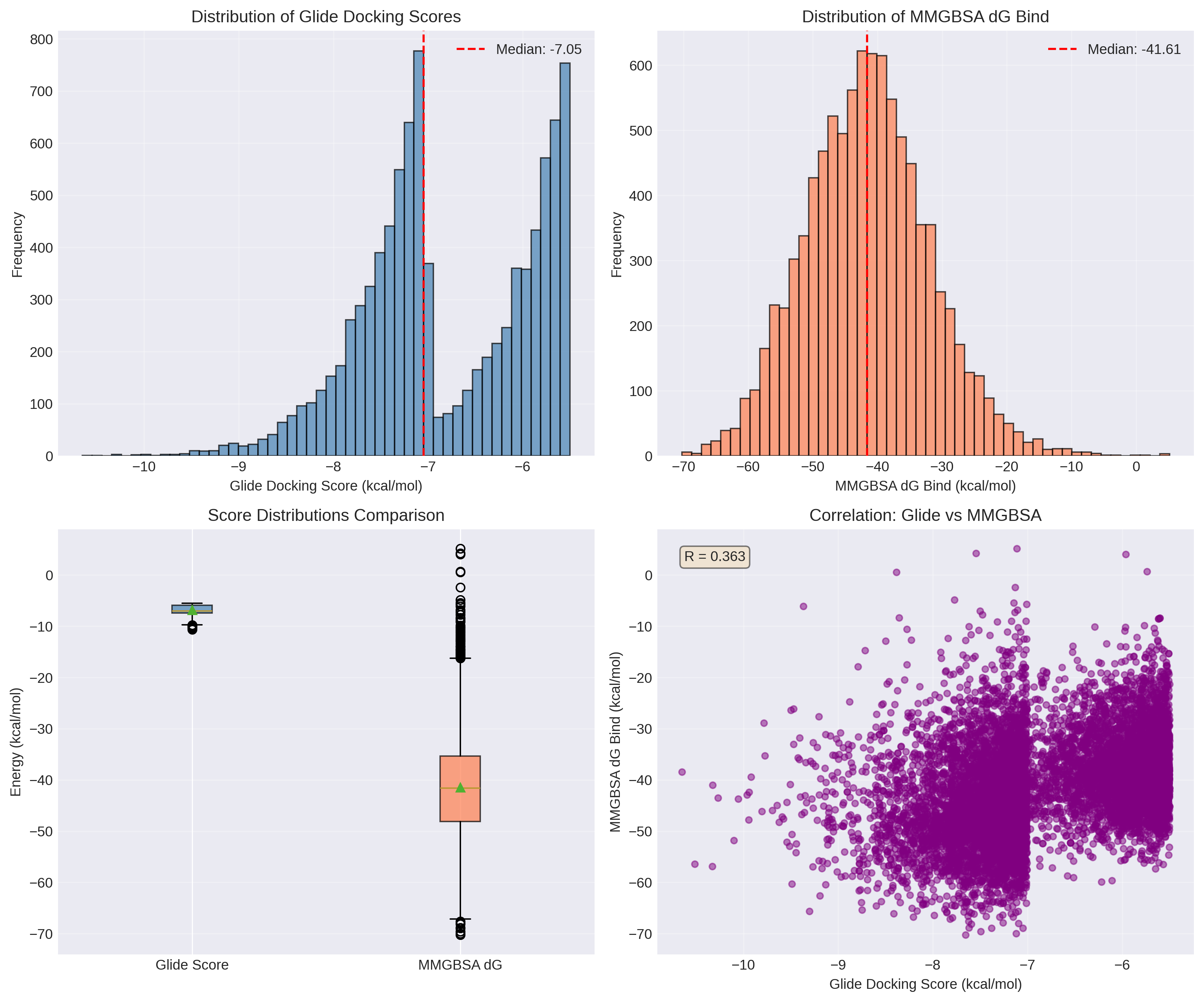

Supplement: Supplementary file 1 — Supplementary material [file mmc1.zip › supplementary file/figures_1_distributions.png]

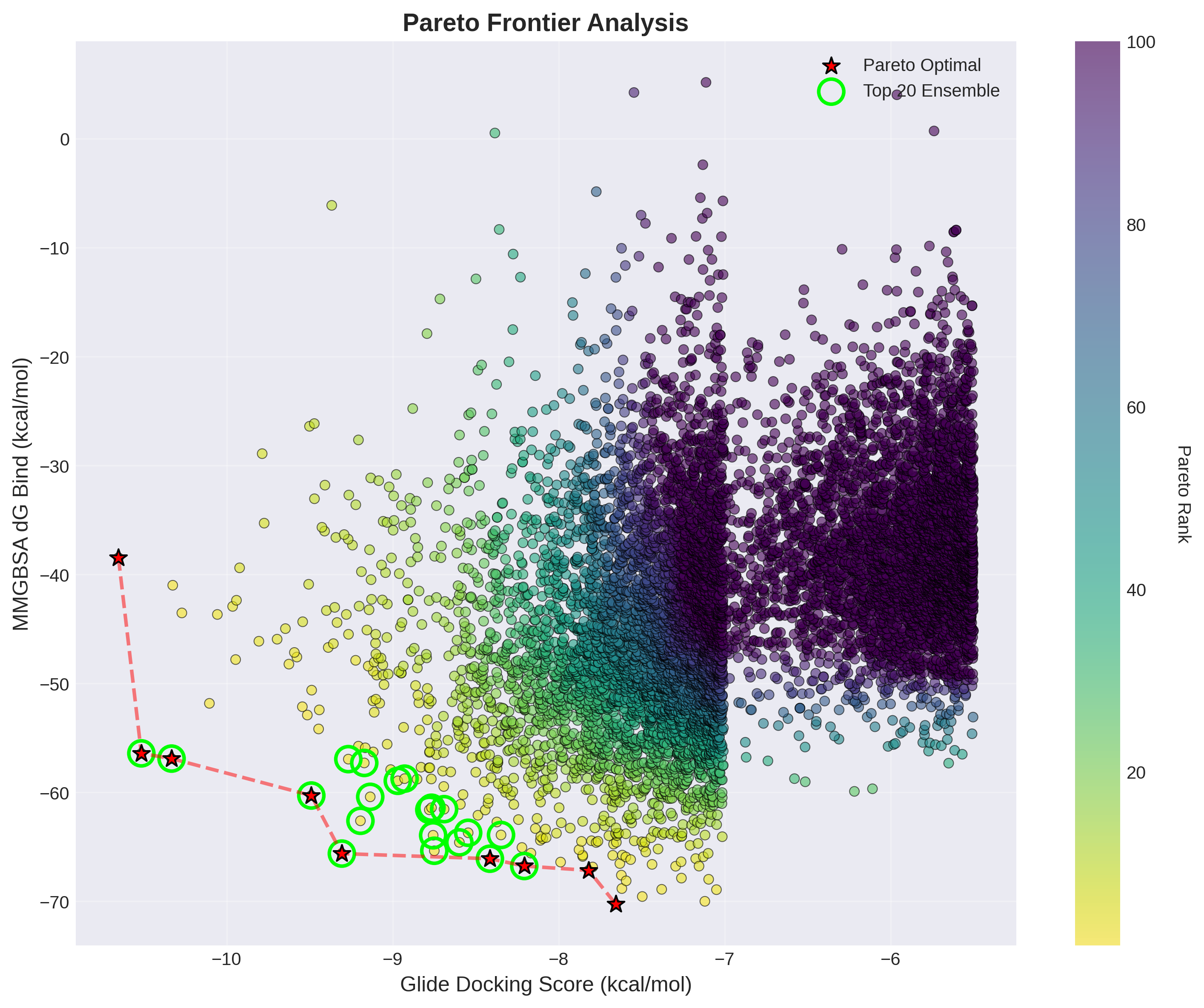

Supplement: Supplementary file 1 — Supplementary material [file mmc1.zip › supplementary file/figures_2_pareto_frontier.png]

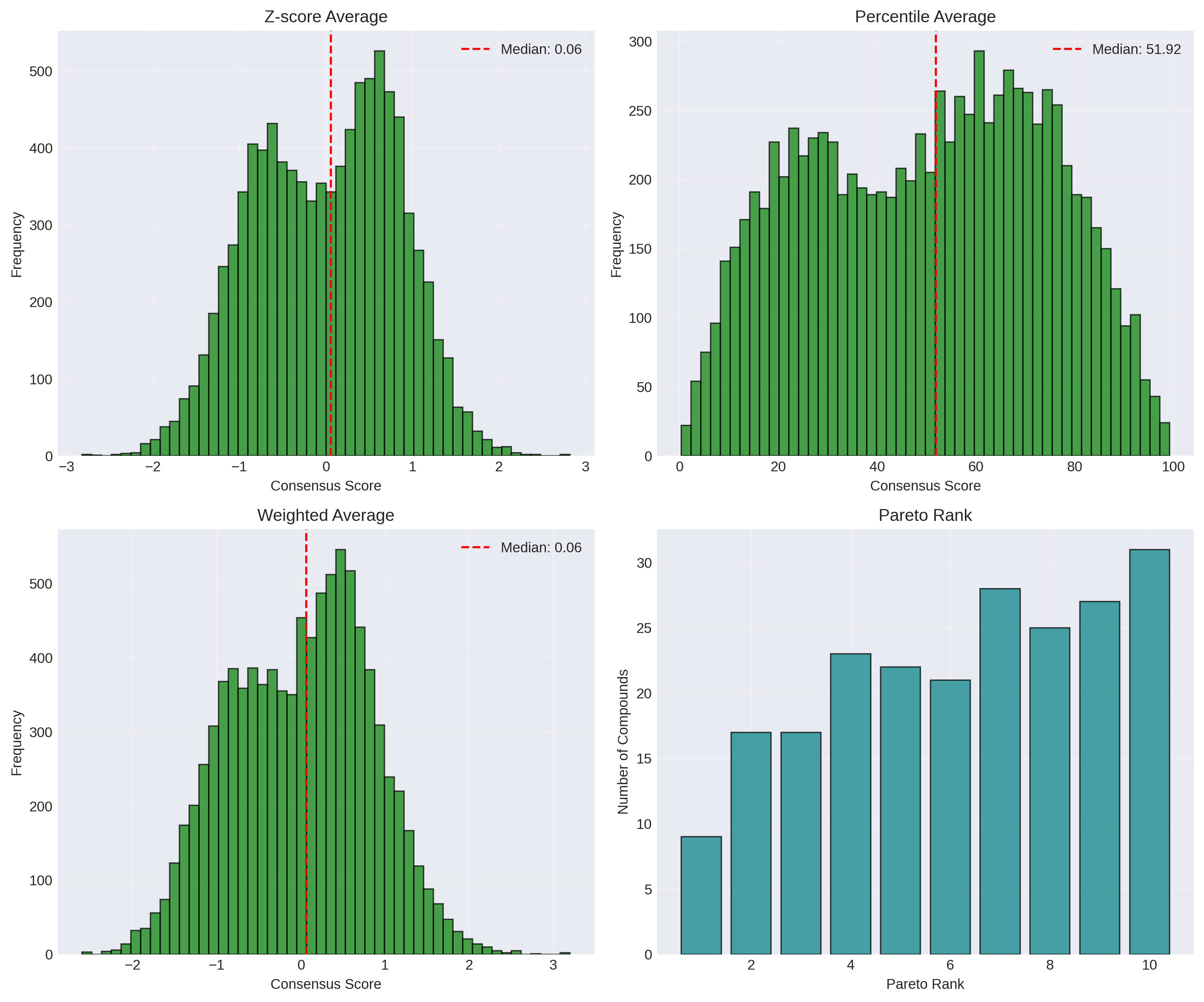

Supplement: Supplementary file 1 — Supplementary material [file mmc1.zip › supplementary file/figures_3_method_comparison.png]

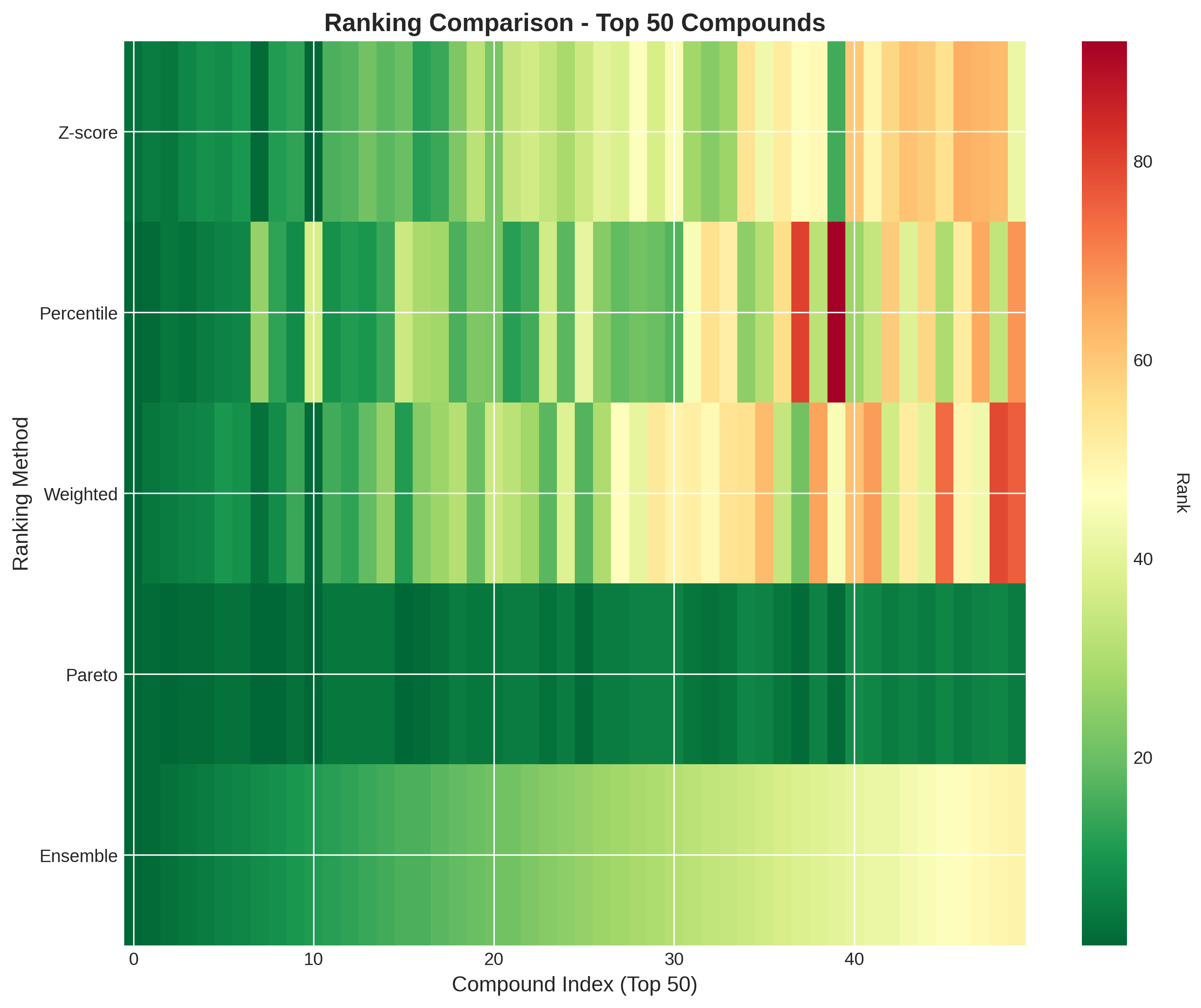

Supplement: Supplementary file 1 — Supplementary material [file mmc1.zip › supplementary file/figures_4_ranking_heatmap.png]

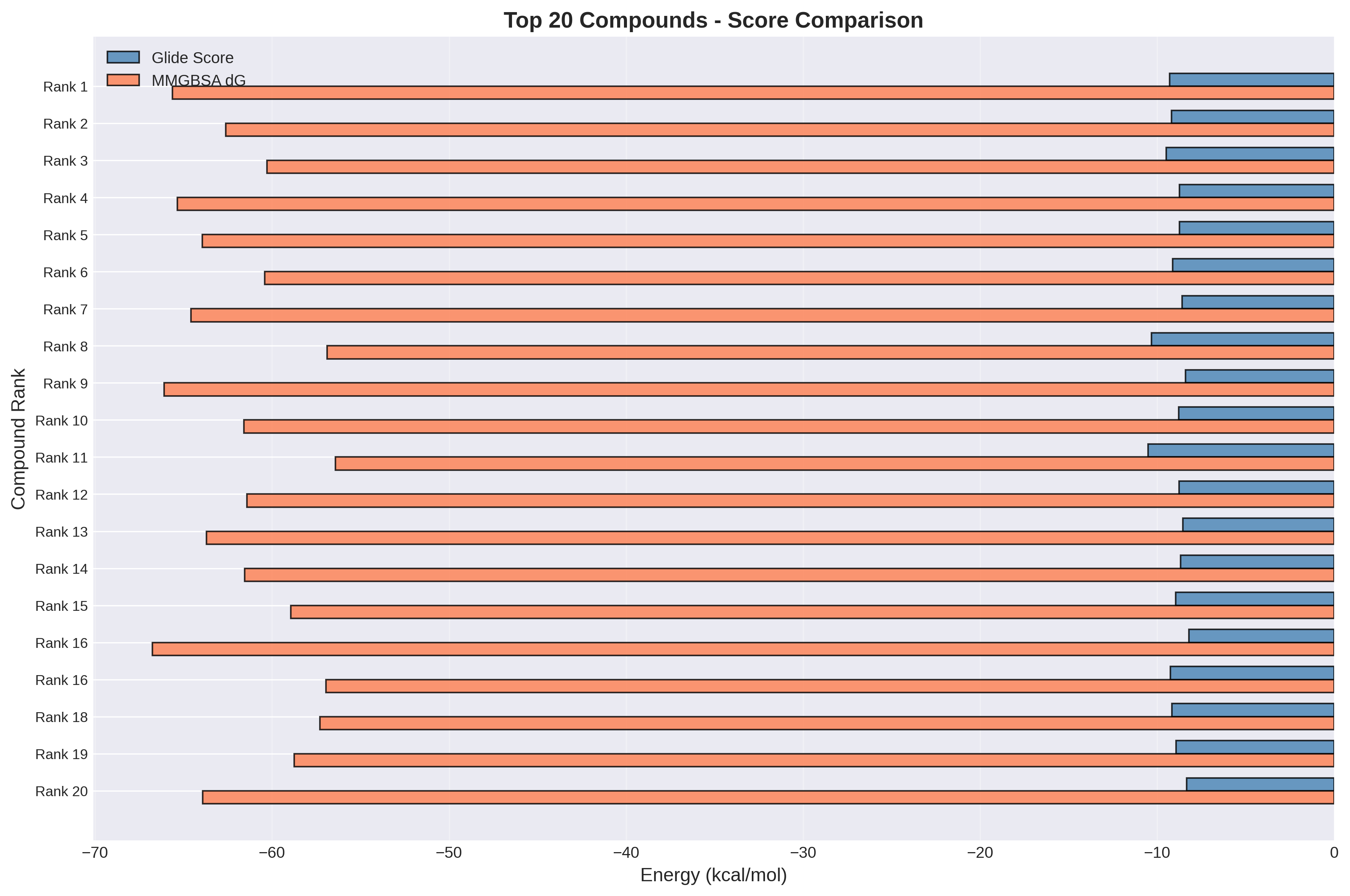

Supplement: Supplementary file 1 — Supplementary material [file mmc1.zip › supplementary file/figures_5_top20_scores.png]

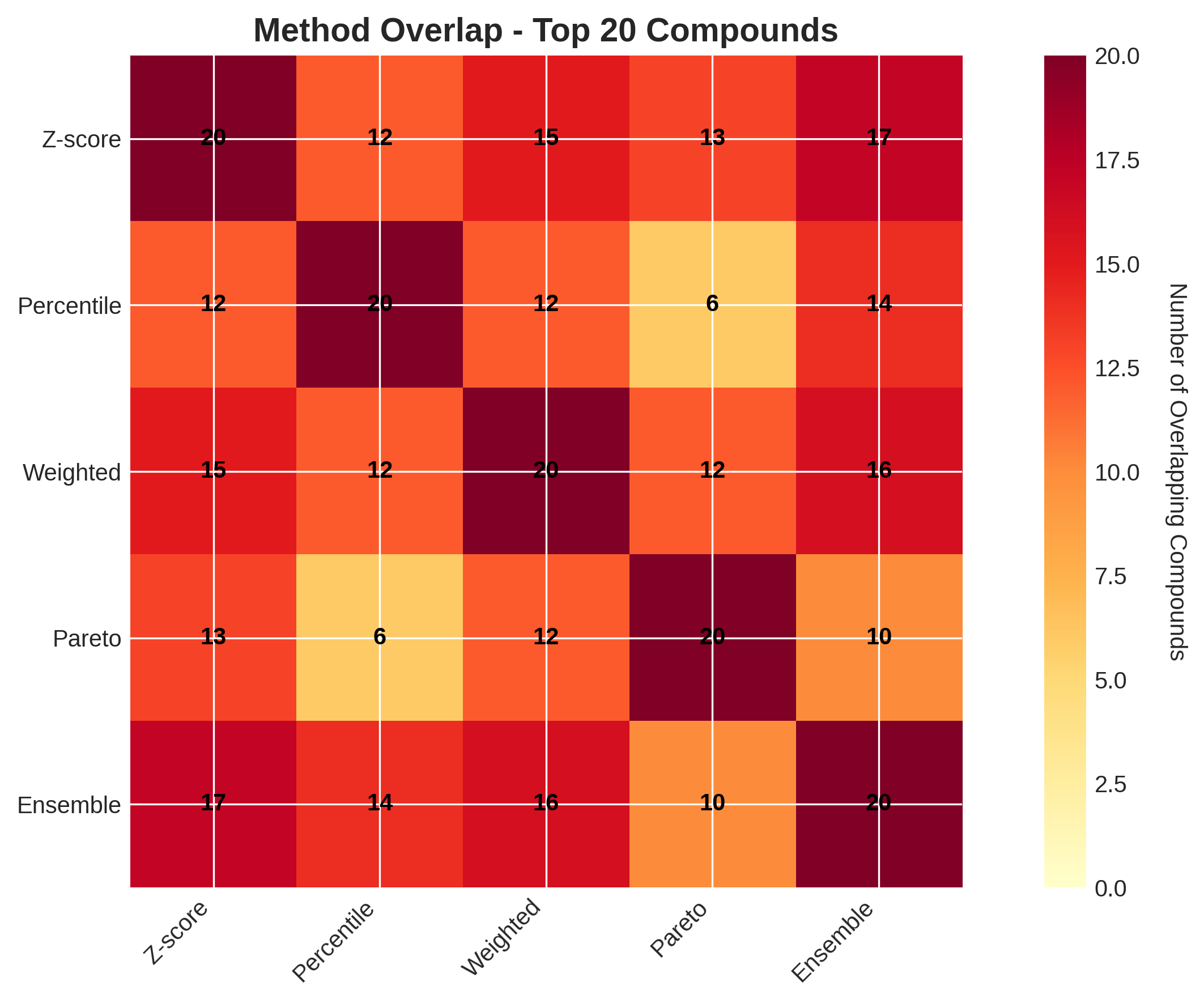

Supplement: Supplementary file 1 — Supplementary material [file mmc1.zip › supplementary file/figures_6_method_overlap.png]
